# The Heidelberg Milestones Communication Approach (MCA) for patients with prognosis <12 months: protocol for a mixed-methods study including a randomized controlled trial

**DOI:** 10.1186/s13063-018-2814-1

**Published:** 2018-08-14

**Authors:** Anja Siegle, Matthias Villalobos, Jasmin Bossert, Katja Krug, Laura Hagelskamp, Johannes Krisam, Violet Handtke, Nicole Deis, Jana Jünger, Michel Wensing, Michael Thomas

**Affiliations:** 1 Department of Thoracic Oncology, Thoraxklinik Heidelberg, Heidelberg University Hospital, Translational Lung Research Center Heidelberg (TLRC-H), German Center for Lung Research (DZL), Röntgenstraße 1, D-69126 Heidelberg, Germany; 2 Department of General Practice and Health Services Research, Heidelberg University Hospital, Im Neuenheimer Feld 130.3, 69120 Heidelberg, Germany; 3 Institute of Medical Biometry and Informatics, Heidelberg University Hospital, Im Neuenheimer Feld 130.3, 69120 Heidelberg, Germany; 4 The German National Institute for State Examinations in Medicine, Pharmacy and Psychotherapy, Große Langgasse 8, 55116 Mainz, Germany; 5 German Cancer Research Center, Im Neuenheimer Feld 280, 69120 Heidelberg, Germany

**Keywords:** Communication, Prognosis, Interprofessional relations, Complex intervention, Multiphase mixed method, Implementation, Palliative care, Prognostic awareness, Randomized controlled trial

## Abstract

**Background:**

The care needs of patients with a limited prognosis (<12 months median) are complex and dynamic. Patients and caregivers must cope with many challenges, including physical symptoms and disabilities, uncertainty. and compromised self-efficacy. Healthcare is often characterized by disruptions in the transition between healthcare providers. The Milestones Communication Approach (MCA) is a structured, proactive, interprofessional concept that involves physicians and nurses and is aimed at providing coherent care across the disease trajectory. This study aims to evaluate these aspects of MCA: (1) the training of healthcare professionals, (2) implementation context and outcomes, (3) patient outcomes, and (4) effects on interprofessional collaboration.

**Methods/design:**

A multiphase mixed-methods design will be used for the study. A total of 100 patients and 120 healthcare professionals in a specialized oncology hospital will be involved. The training outcomes will be documented using a questionnaire. Implementation context and outcomes will be explored through semi-structured interviews and written questionnaires with healthcare professionals and with the training participants and through a content analysis of patient files. Patient outcomes will be assessed in a pragmatic non-blinded randomized controlled trial and in qualitative interviews with patients and caregivers. Trial outcomes are supportive care needs (SCNS-SF34-G), quality of life (SeiQol and Fact-L), depression and anxiety symptoms (PHQ-4), and distress (Distress Thermometer). Qualitative semi-structured interviews on patients’ views will focus on shared decision-making, communication needs, feeling empathy, and further utilization of healthcare services. Interprofessional collaboration will be explored using the UWE-IP-D before the implementation of MCA (t0) and after 3 (t1), 9 (t2), and 12 (t3) months.

**Discussion:**

Using guideline-concordant early palliative care, MCA aims to foster patient-centered communication with shared decision-making and facilitation of advance care planning including end-of-life decisions, thus increasing patient quality of life and decreasing aggressive medical care at the end of life. It is assumed that the communication skills training and interprofessional coaching will improve the communication behavior of healthcare providers and influence team communications and team processes.

**Trial registration:**

German Clinical Trials Register, DRKS00013649 and DRKS00013469. Registered on 22 December 2017.

**Electronic supplementary material:**

The online version of this article (10.1186/s13063-018-2814-1) contains supplementary material, which is available to authorized users.

## Background

The care of patients with a limited prognosis is complex and demanding. Moreover, the process of care is often interrupted by quick transitions between healthcare settings and providers, especially for cancer patients [1], leading to a lack of continuity in care. Throughout the disease trajectory, patients and caregivers must deal with receiving bad news at short intervals. The terminal character of a limited prognosis means that patients need to engage in advance care planning and end-of-life decision-making. Therefore, patients and their caregivers must deal with several dimensions of burden (physical, psychological, social, financial, and spiritual) [2].

The quality of care for patients with advanced cancer hinges, among other factors, on the communication skills of the healthcare professionals involved. Delivering bad news, discussing prognoses and possible disease trajectories (best case and worst case), and preparing others for the end of life are challenging communication topics and demand highly skilled professionals [3]. Yet, patients, caregivers, and the healthcare professionals themselves often perceive these skills to be insufficient [4, 5].

The German National Cancer Plan [6] and other medical societies recommend that healthcare providers should improve their communication skills [6–8]. Indeed, different communication training programs have been developed and evaluated successfully for the medical curriculum [9, 10]. Yet, most of these programs focus on basic communication skills in specific situations [11–14] without considering the whole disease trajectory. Furthermore, communication modules concerning palliative care have been developed but refer mainly to the transition to the best supportive care for the terminally ill [15]. The positive results of trials regarding the early integration of palliative care [16, 17] additionally challenge patient–physician communications as they leave the oncologist with the task of addressing palliative care early in the course of disease.

These trials and others that evaluate the training of communication skills in oncology have shown multiple benefits: improvement of quality of life and advance care planning for patients, improvement in job satisfaction, and decrease of burden on professionals [12, 13, 18–21]. Additionally, different studies on palliative care show that multi-professional approaches are more effective [16, 22–24].

For advanced lung cancer, forward-thinking communication has been described for the German setting [25]. This introduces standardized steps of communication at turning points of treatment, such as first disclosure of diagnosis, disease progression, and transition to best supportive care. Based on findings from interviews with patients and caregivers and focus groups with healthcare professionals, a structured longitudinal concept has been developed: the Heidelberg Milestones Communication Approach (MCA) [5, 26, 27].

MCA is a pro-active, interprofessional concept that involves physicians and nurses and is aimed at providing coherent care that integrates palliative care early and across the disease trajectory. It provides intervention-based communication to develop patient-centered care further through increasingly integrating patient preferences. Metastatic lung cancer has been used as a model disease in developing MCA. Not only is there a limited prognosis with a median survival of less than 12 months but it is also associated with a substantial existential uncertainty and a high symptom burden with detrimental impact on patients and caregivers [27, 28]. However, institutional strategies for implementing longitudinally structured communication concepts such as MCA and knowledge about the effects of implementation are still lacking.

Many interventions in clinical and health services research fail to translate into practice and policy [29]. Implementation processes are complex, take a long time, and are cost intensive [30, 31]. Therefore, the effects and implementation of an intervention need to be evaluated concurrently. Furthermore, implementations are influenced by individual health, professional factors, patient factors, professional interactions, incentives and resources, capacity for organizational change, as well as by social, political, and legal factors [32].

### Aims and objective

This study aims to evaluate these aspects of MCA:

1. training of healthcare professionals
2. implementation context and outcomes
3. patient outcomes
4. effects on interprofessional collaboration.

## Methods/design

### Multiphase mixed-methods design

Since several perspectives (patient, family caregiver, and healthcare professional) and different interventions (communication concept, training, and implementation) at different stages (development, implementation, and evaluation) are relevant, a mixed-methods design has been chosen [33–35]. Our aims contain complex multidimensional processes (social, cognitive, and cultural) and multiple stakeholders (patients, caregivers, and healthcare professionals). To evaluate outcomes, a randomized controlled trial (RCT) and interviews with patients and caregivers are planned. To evaluate processes and context, a combination of quantitative and qualitative methods will be applied [35]. The multiphase mixed-methods design allows a comprehensive understanding of the effects of MCA [34].

### Phase 1: development of the Heidelberg MCA

Our previous studies in phase 1 included a qualitative exploration of patients’ and caregivers’ experiences over the disease trajectory and the assessment of healthcare professionals’ views of a hypothetical structured and forward-thinking communication approach [5, 26, 27]. Based on these results, Heidelberg MCA was developed as a complex intervention: (a) to address the communication needs of patients and caregivers, (b) to improve continuity of care, (c) to improve individual quality of life of patients and their caregivers, (d) to foster shared decision-making including end-of-life decisions, and (e) to enhance communication competencies and team processes of the interprofessional oncology team.

In a second step, we led in-depth interviews with nurses and focus group interviews with physicians at our institution to explore the enablers and barriers of implementation as well as interprofessional collaboration concerning all four components of MCA. Based on the results of these interviews, MCA was adapted and now contains the following components (see Table 1):

**Table 1:** MCA complex intervention components

Intervention component	Subcomponent	Content
Communication training	Two training sessions with simulated patients and video assessment Two training sessions in a clinical setting including individualized feedback Coaching	MCA conversation components Communication skills Attitude Empathy Prognostic awareness
Milestone conversations	Diagnosis	Breaking bad news Treatment options Outlining follow-up and contact with nurse
	Stable phase	Question-prompt-list Prognostic awareness Advance care planning Palliative care needs
	Progression	Question-prompt-list Breaking bad news Prognostic awareness Treatment options Advance care planning Palliative care needs
	Transition to best supportive care	Question-prompt-list Breaking bad news Prognostic awareness Advance care planning Symptom treatment Palliative care needs
Follow-up sessions	Patient is contacted every month by telephone or during a routine clinic visit by a nurse	Palliative care assessment Prognostic awareness Answering patient questions Referrals if necessary
Material	Communication manual for milestone conversations	Content of milestone conversations Training material
	Memory cards	Quick overview of milestone conversations
	Managing symptoms guidebook	Brochure for patients on symptom management
	Question-prompt-list	Questions patients could ask

*MCA* Milestones Communication Approach

1. Interprofessional (physician and nurse) communication training

2. Planned, structured nurse-physician-patient and caregiver conversations at four points within the disease trajectory, the so-called milestone conversations

3. Monthly follow-up sessions for outpatient and ambulatory patients and their caregivers (nurse)

4. Supportive materials (question-prompt-list, managing symptoms guidebook for patients and caregivers, and communication manual and memory cards for nurses and physicians)

For a more detailed description of the intervention, see Additional file 1.

No harm is anticipated from the intervention. If any harm is identified, a referral to psycho-oncology services is possible.

The complete project consists of three phases: (1) further development, (2) implementation, and (3) evaluation. Phase 1 (further development) had a separate ethical approval (ethics committee University of Heidelberg S-139/2017) and is already completed. This study protocol is for phase 2 (implementation) and phase 3 (evaluation). Implementation and evaluation are based on the results of phase 1. During the implementation phase, communication training will be conducted and evaluated. Implementation of MCA will be adapted according to participant experiences. The evaluation phase includes a monocentric non-blinded RCT measuring the effects of MCA in patients with a limited prognosis. In a cohort study, the longitudinal effects on interprofessional collaboration of clinical staff will be observed. Experiences with the concept will be evaluated in qualitative semi-structured interviews with patients and their caregivers (see Table 2).

**Table 2:** Study aim, method, type of data, and outcome

	Study aims	Methods	Participants	Outcomes	Type of data
Phase 2 implementation	Evaluation of communication training	Questionnaire	Physicians and nurses	Training success	Quantitative
	Evaluation of implementation context and outcomes	Interview and focus group interviews	Physicians and nurses	Fidelity and adherence enablers and factors associated with implementation	Qualitative
		Quantitative content analysis of patient electronic files		Adherence	Quantitative
Phase 3 outcome evaluation	Impact of MCA on patient outcomes	Pragmatic RCT using questionnaire	Patients and caregivers	Supportive care needs Patient quality of life Patient distress	Quantitative
		Semi-structured interviews	Patients and caregivers	Patient reported outcomes	Qualitative
	Evaluation of effects on interprofessional collaboration	Questionnaire	Healthcare professionals	Attitudes towards interprofessional collaboration	Quantitative

*MCA* Milestones Communication Approach, *RCT* randomized controlled trial

To implement MCA, it is important to understand how MCA works within a real-life clinical setting. This implementation implies working with healthcare professionals who will be affected by MCA and investigating the context of the intervention. A hospital context is not a fixed organizational structure but an unstable, unfolding process [36]. Thus, implementation of MCA will be conducted using a plan-do-check-act (PDCA) cycle [37].

- Plan: Plan communication training and a theory-based implementation
- Do: Conduct communication training and make first attempts at using MCA
- Check: Investigate experiences with trained nurses and physicians
- Act: Reintegrate evaluation results into everyday MCA practice

If necessary, the PDCA cycle will be used several times. This approach ensures that the adaptability, usability, and feasibility of MCA are incorporated [32].

#### Setting

The project will be conducted at the Department of Thoracic Oncology, University Hospital Heidelberg, which is one of the largest lung cancer centers in Germany. Every year, about 600 patients are newly diagnosed with metastatic lung cancer. The physicians involved in the project are oncologists and residents in advanced oncology, working mainly at the outpatient clinic. Their daily consultations include the first disclosure of diagnosis and prognosis, disclosure of progression, and transition to best supportive care (breaking bad news). The nurses have working experience in oncology and palliative care in both inpatient and outpatient settings.

### Phase 2: implementation

#### Evaluation of communication training outcomes

##### Questionnaire for training evaluation

To explore training, acceptance level 1 (reaction) and level 2 (learning) of the Kirkpatrick model will be evaluated [38]. The sample includes five nurses and five physicians, who will receive the training. To integrate MCA communication content, participants will complete a self-assessment questionnaire with 26 items on acquisition of intended knowledge, skills, attitude, confidence, and commitment based on participation in the training and relevance to participants’ work. Items are rated on a five-point Likert scale ranging from agree completely (1), agree (2), neither agree nor disagree, disagree (4) to disagree completely (5). In addition, there are four open-ended questions on what participants liked best or least about the training, their overall impression, and what would help to enhance their communication skills. Training participants (*n* = 10) will be invited to complete the questionnaire after all four training sessions. The questionnaires will be analyzed using descriptive statistics, including mean, median, standard deviation, and range. Thematic analysis will be used to evaluate open-ended questions [39]. The results of each training session will be used to plan and improve the next training session.

#### Evaluation of implementation context and outcomes

##### Interviews to evaluate implementation

Interviews and focus group interviews with training participants will be conducted between the training sessions to explore participants’ experiences with and transfer of MCA into everyday practice. A topic guide derived from the literature and our previous studies will be developed to determine experiences and context factors that influence training, communication, implementation, and collaboration [40]. The main issues raised in the interviews will be recorded on a protocol sheet, summarized, and the results fed back by the researchers to the communication training and implementation team [41]. The results will be used to improve the next training sessions, to enhance implementation in everyday practice, and to adapt both MCA materials (communication manual, memory cards, question-prompt-list, and brochure) and the implementation plan following the PDCA cycle.

##### Quantitative content analysis of patient files

From kickoff, nurses will document excerpts from the milestone conversations and follow-up calls in patient electronic files. Excerpts written 6 months after kickoff (*n* = 50) from the milestone conversations and (*n* = 50) from the follow-up calls will be analyzed for adherence to the MCA concept, shared decision-making, prognostic awareness, and documented topics [41]. The file content will be analyzed using a fidelity checklist of essential topics based on the MCA manual. Data will be entered into SPSS and descriptive statistics will be produced. Binary and continuous variables will be evaluated by mean, median, minimum, and maximum, and categorical variables using absolute and relative frequencies [41]. The results will help to determine to what extent the developers’ intentions regarding training contents and adherence to the communication manual are seen in practice.

### Phase 3: outcome evaluation

#### Impact of MCA on patient outcomes

The effects of MCA on patients and caregivers will be determined in a RCT. Their experiences with the concept will be assessed in semi-structured interviews. The effects on interprofessional collaboration will be evaluated in a longitudinal observational study with healthcare professionals.

#### Effects on patients and caregivers (pragmatic RCT)

In the RCT, the effects of MCA on patients and caregivers will be assessed and evaluated with a focus on shared decision-making and on early and proactive advance care planning. Patients and their caregivers will report on daily life activities and the identification and treatment of palliative care needs. The impact of the concept regarding empathy, quality of life, and distress in patients with metastatic lung cancer will also be examined. Furthermore, needs-based use of services and the impact of further contacts in the German healthcare system will be assessed.

##### Primary outcome

The primary outcome is the effect of MCA on meeting patients’ information needs measured using the health system and information needs dimension of the Supportive Care Needs Survey: Short Form for Patients (German version) (SCNS-SF34-G).

##### Secondary outcomes

The secondary outcomes are the effects of MCA on perceived empathy, quality of life, and distress in patients with metastatic lung cancer and a limited prognosis, needs-based use of health services, and further contacts with healthcare professionals in the German healthcare system.

##### Sample and sample size

The study will consist of 100 patients and 100 caregivers named by the patients. It will include patients with newly diagnosed metastatic lung cancer (stage IV) who are at least 18 years old, able to give consent, have a good knowledge of the German language, and are willing to participate. Participating caregivers also must be at least 18 years old, have a good understanding of the German language, be willing to participate, and give consent.

##### Data collection

**Fig. 1:**
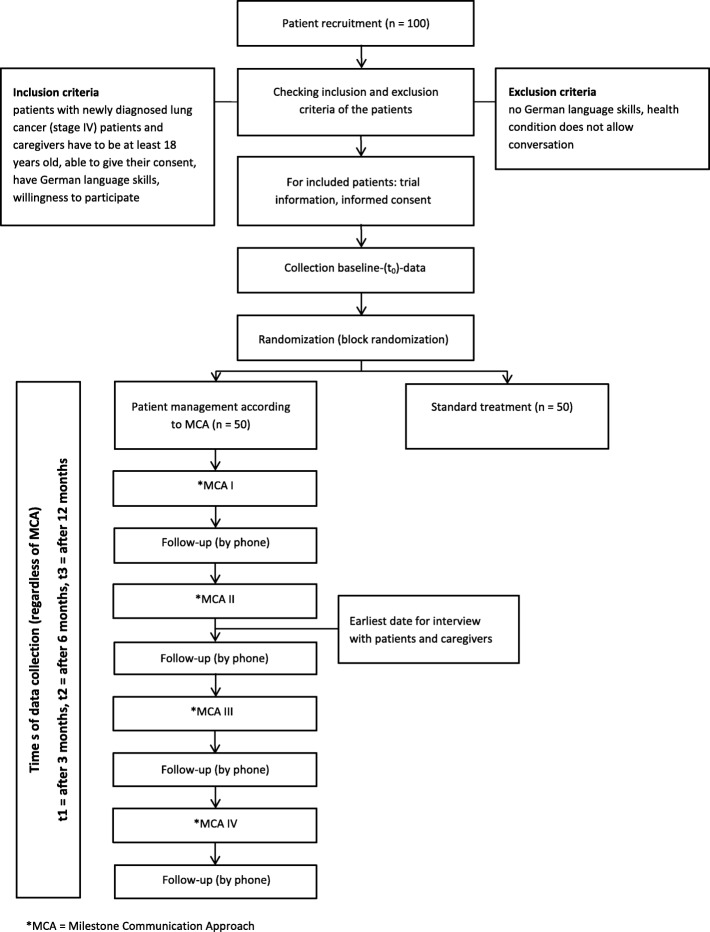
Flow diagram for patient recruitment

**Fig. 2:**
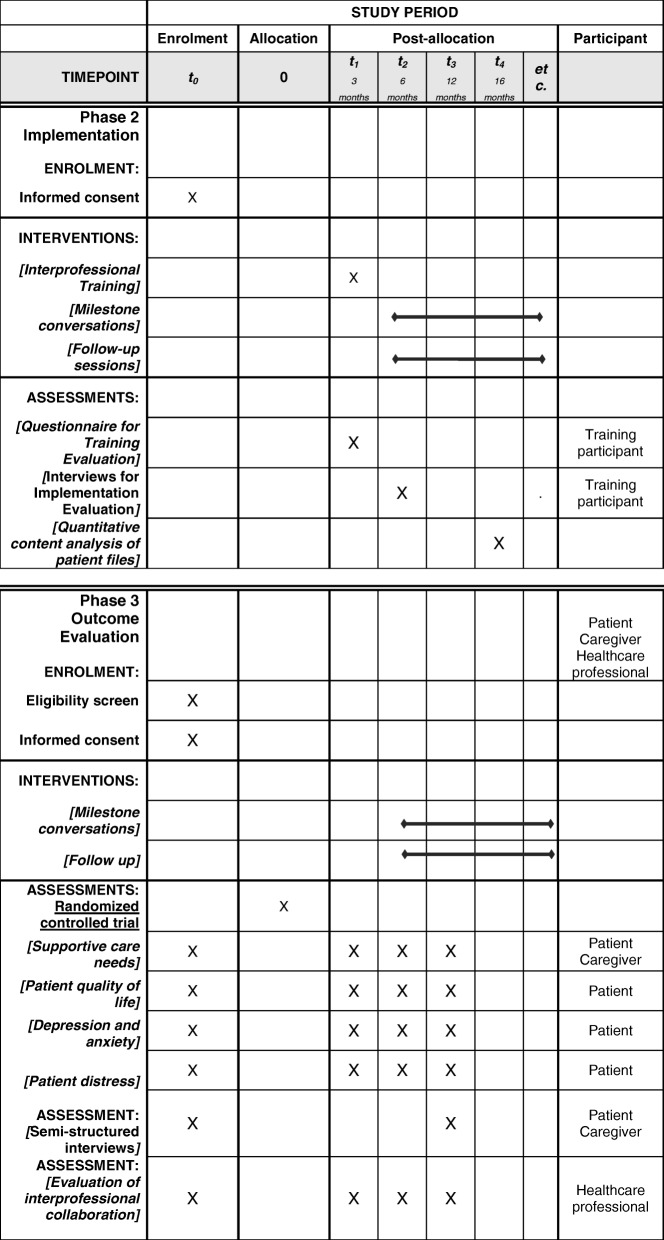
Study plan

Recruitment of patients and caregivers will take place at the Department of Thoracic Oncology, University Hospital Heidelberg. Patients and caregivers will be approached by a study nurse who works at the department. If patients and caregivers are interested in the project, they will receive oral and written information about the study. After giving consent, baseline (t0) questionnaire data will be collected before patients are randomly assigned to a group. The first milestone conversation (tandem or standard communication with a physician) will then take place. For the follow-up, questionnaires will be distributed after 3 (t1), 6 (t2), and 12 (t3) months (Fig. 1). Study plan according to SPIRIT-checklist see Fig. 2 and Additional file 2. Patient documents will be checked by a study nurse for adverse events. No adverse events are anticipated.

##### Randomization procedure

The randomization into the groups (intervention and control) will occur in the ratio 1:1. Block randomization will be performed to ensure equal-sized groups. The randomization will be performed using sealed opaque randomization envelopes, which will be provided by the Institute of Medical Biometry and Informatics Heidelberg.

##### Instruments

All instruments are validated in German. Table 3 provides an overview of the assessment instruments. The supportive care needs of patients will be assessed using SCNS-SF34-G. The questionnaire comprises 34 items and covers five domains: (1) health system and information, (2) psychological, (3) physical and daily living, (4) patient care and support, and (5) sexual needs. Patients indicate on a five-point scale if and to what degree they are in need of support (1 not applicable; 2 no need, satisfied; 3 low need; 4 moderate need; or 5 high need) [42]. Subscale scores are obtained by calculating the mean of scale items. The higher the subscale score, the higher the need for support in the respective domain [43].

**Table 3:** Outcomes and instruments

Outcome	Instrument
Supportive care needs	SCNS-SF34-G (for patients) [42] SCNS-P&C-G (for caregivers) [44]
Patient quality of life	SEIQoL-Q [46] FACT-G, FACT-L [47, 48]
Depression and anxiety	PHQ-4 [47]
Patient distress	Distress thermometer [56]

*FACT-G* Functional Assessment of Chronic Illness Therapy: general, *FACT-L* Functional, Assessment of Chronic Illness Therapy: lung cancer, *PHQ-4* Patient Health Questionnaire: short form (four questions), *SCNS-P&C-G* Supportive Care Needs Survey for Partners and Caregivers (German version), *SCNS-SF34- G* Supportive Care Needs Survey: Short Form for Patients (German version), *SEIQoL-Q* Schedule for the Evaluation of Individual Quality of Life

The supportive care needs of caregivers will be measured using the German version of the Supportive Care Needs Survey for Partners and Caregivers (SCNS-P&C-G). The multidimensional questionnaire consists of 45 items in four domains: (1) healthcare service needs, (2) psychological and emotional needs, (3) work and social needs and (4) information needs, which are assessed on a five-point scale (1 I have no problems, 2 I am already supported, 3 low need, 4 moderate need, and 5 high need). Answers 1 and 2 are grouped together so that 1 stands for “there is no need for support.” For supportive care needs domains, the mean of the respective items will be calculated, ranging from 1 to 5 [44], and standardized on a 0–100 scale [45].

A patient’s quality of life will be assessed using the Schedule for the Evaluation of Individual Quality of Life (SEIQoL-Q) [46], the Functional Assessment of Chronic Illness Therapy (basic module, FACT-G, and additional questions on lung diseases, FACT-L) [47, 48]. The Patient Health Questionnaire (PHQ-4) [49] will be used to assess depression and anxiety in patients. The SEIQoL-Q measures each patient’s quality of life by choosing, rating, and weighting five domains that patients consider important, such as family, health, or social life/other relations [50]. The five-point scale ranges from not at all (0) to extremely (100) important. Following this, patients must evaluate their overall satisfaction with the areas of life on the same scale [51].

FACT-G consists of 27 items in four domains: (1) physical well-being, (2) social well-being, (3) emotional well-being, and (4) functional well-being. All domains, except emotional well-being, consist of seven items, each with a score in the range 0–28. Emotional well-being contains six items and has a score range of 0–24. For all questions in FACT-G, a five-point rating scale from 0 to 4 (0 not at all, 1 a little bit, 2 somewhat, 3 quite a bit, and 4 very much) is used. The total score for FACT-G is calculated as the sum of the four subscale scores, provided that the overall item response is at least 80% (i.e., at least 22 of the 27 items were answered) and takes values between 0 and 108. Negatively expressed items are reverse scored prior to summing so that higher subscale and total scores indicate a better quality of life [47, 48, 52]. FACT-L contains the four domains of FACT-G and one lung cancer symptom-specific subscale. The seven items of the lung cancer subscale assess patient-reported symptoms, like shortness of breath and loss of weight. FACT-L is also rated on a five-point Likert-scale ranging from 0 (not at all) to 4 (very much) with a score ranging from 0 to 28 [53].

PHQ-4 is an ultra-brief self-report questionnaire that contains a two-item depression scale (PHQ-2) and a two-item anxiety scale (GAD-2) [49]. Patients assess how many times over the past 2 weeks they have felt a loss of interest and happiness, depression, melancholy or hopelessness, nervousness, anxiety or concern, and restlessness. The ordinal-scaled answer options are not at all (0), several days (1), more than half the day (2), and nearly every day (3) [54]. The total score for PHQ-4 ranges from 0 to 12, with categories of psychological distress being none (0–2), mild (3–5), moderate (6–8), and severe (9–12) [55].

Patient distress will be measured using the NCCN Distress Thermometer. This assessment technique was developed by the National Comprehensive Cancer Network (NCCN) and is a brief self-reported screening instrument for recording psycho-social stress in oncological patients. It consists of a scale from 0 to 10 and a problem list. A score of 5 or higher indicates that a patient is conspicuously stressed and needs assistance [56]. If the burden is low (0–4), no additional professional support is required [56].

##### Data analysis

All baseline patient characteristics will be analyzed descriptively. Continuous variables will be described as means with standard deviation or as medians with interquartile range, minimum, and maximum. Categorical variables will be described in absolute and relative frequencies.

The sum of the health system and information dimension score of SCNS-SF34-G is defined as the primary outcome of the RCT. The primary outcome at t1 will be analyzed using a linear model in which the outcome value is included as dependent variable and the respective baseline value and the treatment group as independent variables. Missing values in the primary outcome will be replaced by multiple imputation, which consider treatment the group and primary outcomes at t0 as independent variables using the fully conditional specification method [57]. A complete case analysis will be performed as an additional sensitivity analysis. The respective parameter estimates will be reported together with *p* values and 95% confidence intervals. The analysis of the primary outcomes at t2 and t3 will be done analogously to the primary linear model. The analyses of the secondary outcomes will be performed using linear (for continuous outcomes) or generalized linear models (for binary outcomes). The models include the outcome value at follow-up time t1, t2, or t3 as the dependent variable. The respective parameter estimates will be reported alongside descriptive *p* values and 95% confidence intervals. *p* values smaller than 0.05 will be considered statistically significant. Analysis will be done using the statistical software SAS v9.4 (SAS Institute, Cary, NC).

##### Power calculation

With a sample size of *n* = 100, a difference of 5 points on a scale from 0 to 100 for the primary outcome can be shown using a two-sided *t*-test at a significance level of α = 0.05 with a power of 1 – ß = 0.85, assuming a standard deviation of σ = 7.4 [58]. A dropout rate of 18.5% is taken into account. Prognoses of metastatic lung cancer and the experiences at the Department of Thoracic Oncology, University Hospital Heidelberg have shown that 82.5% of the patients are still alive after 3 months and 75% are still alive after 6 months. It can be assumed that the additional variance explained by the inclusion of the baseline value as a covariate will lead to increased power. The sample size calculation was performed using SAS v9.4 (SAS Institute, Cary, NC).

#### Effects on patients and caregivers (semi-structured interviews)

Experiences with the MCA concept regarding empathy and shared decision-making will be evaluated through interviews with patients and caregivers.

##### Sample and sample size

Patients and caregivers who will experience MCA will be approached in the hospital setting. Patients and caregivers must be over 18 years old and able to understand and speak German well. They will be asked to participate and receive a written invitation to the study, including the background information, participation details, and informed consent form. About 12 patients and 12 caregivers will be interviewed, possibly more, until data saturation is reached.

##### Data collection

Semi-structured interviews will be conducted at the Department of Thoracic Oncology, University Hospital Heidelberg, after at least two milestone conversations have taken place. A theory-based topic guide with open-ended questions will be used. Interviews will be digitally recorded and transcribed verbatim [59].

##### Data analysis

Interviews will be analyzed using qualitative content analysis as described by Mayring [60]. The qualitative content analysis consists of nine steps for analyzing texts: (1) determination of the material, (2) analysis of the original situation, (3) formal characteristics of the material, (4) determination of the direction of the analysis, (5) theoretical differentiation of the question, (6) determination of the analysis techniques and definition of the concrete process model, (7) definition of the analysis units, (8) analysis steps using the category system (abstract explication and structuring) and review of the category system of theory and material, and (9) interpretation of the results in the direction of the question and application of content-analytical quality criteria. The Mayring concept is based on reducing the initial material and is, therefore, suitable for large amounts of data and systematic textual processing, as is the case in this work. Although it cannot be used for an explorative-interpretive analysis [61], the concept is well suited to answering the questions posed in this project.

#### Evaluation of interprofessional collaboration

Clinical employees will assess the effects of the approach on interprofessional collaboration and on the understanding of their own role within their team.

##### Sample and sample size

All 120 team members of the medical, nursing, administration, psycho-social and therapeutic professions at the Department of Thoracic Oncology, University Hospital Heidelberg will receive oral and written information on the MCA project with the request to participate. Participants will then receive a written invitation to participate in the study, including the background information, participation details, and informed consent form.

##### Data collection

With the invitation, participants are asked to complete an attached questionnaire prior to the first training within the MCA project (t0), directly after the implementation phase (t1), and 6 (t2) and 12 (t3) months after the implementation phase.

##### Instrument

The German version of the University of the West of England Interprofessional Questionnaire (UWE-IP-D) will be used to assess interprofessional collaboration and attitudes on teamwork of the healthcare professionals [62, 63]. UWE-IP-D is a self-report instrument consisting of 34 items in a set of four scales addressing different themes. It is administered at different stages in training and education. We used three of the four scales: communication and teamwork scale, interprofessional interaction scale, and interprofessional relationships scale. Communication and teamwork items will be measured on a four-point Likert scale (1 strongly agree, 2 agree, 3 disagree, and 4 strongly disagree) leading to sum scores between 9 and 36, with scores 9–20, 21–25, and 26–36, respectively indicating a positive, neutral, or negative self-assessment of communication and teamwork skills. Interprofessional interaction and interprofessional relationship items are assessed on a five-point Likert scale (1 strongly agree, 2 agree, 3 undecided, 4 disagree, and 5 strongly disagree). The interprofessional interaction scale takes sum scores between 9 and 45, with scores 9–22, 23–31, and 32–45, respectively, indicating positive, neutral, and negative perceptions of interprofessional interaction. Sum scores on the interprofessional relationships scale vary between 8 and 40, with scores 8–20, 21–27, and 28–40, respectively, indicating positive, neutral, and negative attitudes towards the respondent’s own interprofessional relationships.

Additionally, healthcare professionals will report gender and profession (nursing, medical, psycho-social, therapeutic, administrative, or other allied healthcare profession).

##### Data analysis

All the characteristics of the healthcare professionals will be analyzed descriptively. Categorical variables are given as absolute and relative frequencies. UWE-IP-D sum scores will be described as means with standard deviation and as median with interquartile range, minimum, and maximum. Differences between assessments will be analyzed using repeated-measures analysis of variance (ANOVA).

### Data integration

A framework analysis will be used to integrate the data from the quantitative and qualitative research collected from different teams of researchers [64]. The following steps are taken: familiarization with the material, identifying a thematic framework, indexing, charting, mapping, and interpretation [64, 65]. Emerging themes will be related to a priori identified domains [32, 64, 65]. Findings from all the phases will be merged using an integrative analysis [35].

### Ethical aspects

Written informed consent will be obtained from each participant. Ethical approval has been given by the Ethics Committee of the University Hospital Heidelberg (S-561/2017). Participants can withdraw their consent at any time. Only investigators will have access to the final trial dataset. There are no contractual agreements that limit such access. Personal information and the confidentiality, coding, security, and storage of the data are in line with German privacy protection law (Bundesdatenschutzgesetz or BDSG) and the privacy policy of University Hospital Heidelberg.

## Discussion

In routine practice, the care of and communication with patients with a limited prognosis is still characterized by discontinuity and lack of coordination. Inadequate communication makes coping with the realities and choices of a complex incurable disease more difficult. A longitudinal communication approach with a focus on the disease trajectory that comprises specific milestones can serve to integrate early palliative care into routine practice and can facilitate care that is individualized to patients’ needs and preferences. While there are international guidelines for advanced cancer care [3, 7], comprehensive implementation strategies are still lacking. As many structural and organizational aspects of national healthcare systems differ substantially, any transfer of guidelines to the German healthcare system should happen according to the specific situation and needs in Germany.

The MCA project includes the conceptualization of a communication strategy that focuses on a process in which patients and their caregivers are equally involved. The improved communication support should foster prognostic awareness and therefore, facilitate advance care planning and end-of-life decision-making.

### Expected impact

The structured integrated tandem approach (physician and nurse) with interprofessional training and coaching is innovative. Consequently, the strengthening of interprofessional collaborations can be expected. The stepwise approach of the MCA project supports the communication skills and strategies of interprofessional healthcare teams involved in the care of patients with a limited prognosis. It will also enhance patients’ quality of life and improve the continuity and coordination of care. The mixed-methods design will provide a detailed insight into this context, including the perspectives of professionals, patients, and their caregivers.

### Limitations and strengths

A strength of this study is that it simultaneously embeds implementation and evaluation, using a strong design (randomized trial) to assess outcomes. The mixed-methods process evaluation will help in gaining a comprehensive understanding of what works and why. The weaknesses of the quantitative methods are balanced by the qualitative methods and vice versa. This exploration of a complex intervention is characterized by multiple methods and multiple stakeholders in two phases. This complexity requires a mix of researchers from different research fields with experience in qualitative and quantitative research. The phases of the MCA project build upon each other. Determining the degree to which MCA is implemented in a real-world setting will give a better understanding of the measured effects. Adaption of the interventions throughout the implementation will ensure the practicability and transferability into everyday practice.

The sample sizes of the different investigations are small but sufficient for an initial exploration of MCA in a real-world setting. However, for a definitive assessment of the effectiveness of MCA, a larger multicenter RCT is necessary. Since the same physicians will be treating patients in the intervention arm as well as the control arm, there is a potential for bias and cross-contamination between the trial arms. However, the nurses participating in the milestone conversations and offering follow-up for patients and caregivers are exclusively in the intervention arm. Our primary end point is a subjective (non-blind) patient-reported outcome, so there is a potential for bias since patients know they are in a trial and they know the purpose of the trial.

Implementing MCA and identifying relevant determinants will be explored using the example of metastatic lung cancer patients. Positive effects will be used to implement an applicable version of the concept to other medical facilities and patients with a limited prognosis.

### Trial status

The beginning of recruitment for phase 2 and phase 3 is planned for May 2018, and will be completed approximately by November 2019.

## Additional files

Additional file 1: Description of the intervention. (PDF 293 kb)

Additional file 2: SPIRIT 2013 Checklist: Recommended items to address in a clinical trial protocol and related documents*. (PDF 144 kb)

Additional file 3: Confirmation Approval. (PDF 717 kb)

## Abbreviations

ANOVA: Analysis of variance
BDSG: Bundesdatenschutzgesetz
FACT-G: Functional Assessment of Chronic Illness Therapy: general
FACT-L: Functional Assessment of Chronic Illness Therapy: lung cancer
FCS: Fully Conditional Specification
GAD-2: Generalized Anxiety Disorder: short form (two questions)
MCA: Milestones Communication Approach
MI: Multiple imputation
NCCN: National Comprehensive Cancer Network
NCT: National Center for Tumor Diseases
PDCA: Plan-do-check-act
PHQ-2: Patient Health Questionnaire: short form (depression)
PHQ-4: Patient Health Questionnaire: short form (four questions)
RCT: Randomized controlled trial
SAS: Statistical Analysis Systems
SCNS-P&C-G: Supportive Care Needs Survey for Partners and Caregivers
SCNS-SF34- G: Supportive Care Needs Survey: Short Form for Patients (German version)
SEIQoL-Q: Schedule for the Evaluation of Individual Quality of Life
SPSS: Statistical Package for the Social Sciences
UWE-IP- D: University of the West of England Interprofessional Questionnaire: Interprofessional
