# Supplementary material for: The Heidelberg Milestones Communication Approach (MCA) for patients with prognosis <12 months: protocol for a mixed-methods study including a randomized controlled trial
Source: Trials. 2018 Aug 14;19:438. doi: 10.1186/s13063-018-2814-1 (PMC6092809; doi:10.1186/s13063-018-2814-1)
Supplement: Supplementary file 1 — Description of the intervention. (PDF 293 kb) [file 13063_2018_2814_MOESM1_ESM.pdf]

Intervention description (TiDieR)

1 Brief name:

Heidelberg milestones communication approach (MCA)

2 Why:

There is a need for concepts on establishing communication continuity over the disease trajectory, offering advance care planning and integrating early palliative care in patients with a limited prognosis (< 12 months).

3 What – Materials:

3.1 Interprofessional communication training

- *Milestone conversation manual for physicians and nurses* (in German): Detailed description of content of planned, structured nurse-physician-patient consultations including caregivers (with rationale, objectives, definitions and procedures)
- *Memory cards for physicians and nurses* (in German): pocket-sized overview of essentials of MCA conversation manual.
- *Training materials* (in German): Description of communication techniques and exercises for all four training sessions.
- *Training observation checklist* (in German): List of essential components trainers use to evaluate physician-nurse communication exercises during the training and coaching.

3.2 Milestone (nurse-physician-patient) conversation

- *Question-Prompt-List for patients*: Questions selected and translated into German [1-3].
- *Brochure for patients*: Managing lung cancer symptoms (developed by the Roy Castle Lung Cancer Foundation), translated into German, adapted to the German health care system.
- *Milestone conversation file* (for other members of the professional team) with the content of the consultations and reactions of patient and caregiver.

3.3 Follow-up session

- *Follow-up sessions manual for nurses* (in German): Detailed description of the content of follow-up phone calls or meetings for nurses (including rationale, objectives, possible questions to ask, definitions and procedures).
- *Follow-up chart* (in German) with the content of follow-up sessions and the stated needs and reactions of patients and/or caregivers.
- *Integrated palliative outcome Scale (IPOS)* – (German Version): Instrument to measure palliative care needs of patients in relation to symptoms but also extending to information needs, practical concerns, anxiety or low mood, family anxieties and overall feeling of being at peace [4].

4 What – Procedures

The MCA intervention consists of three components: interprofessional communication training, milestone (nurse-physician-patient-caregiver) conversation and follow-up nurse sessions.

4.1 Interprofessional communication training

The interprofessional communication training has been developed for physicians and nurses with experience in patient care.

5 Who

Three trainers are developing and conducting the training: One trainer from the medical field with clinical medical experience, communication experience and interprofessional training and two trainers with experience in didactics, tutor education in the medical field and in physician-patient-communication training.

6 How

Training 1 has been developed by the trainers based on our previous studies, communication theories and the content of the milestone conversation manual for physicians and nurses. Training 2, 3 and 4 are being developed continuously using results of interim evaluations concerning the experiences made and needs stated by the participants as well as organizational aspects. Training 1 (milestone conversations 1 and 2) and training 3 (milestone conversations 3 and 4) comprise topics on breaking bad news, physician-patient communication, interprofessional communication, prognostic awareness [5], hope, Spikes [6], NURSE and shared decision making. The theoretical input of training sessions 1 and 3 have been trained with simulated patients. The conversations with the simulated patients will be video analyzed and individualized feedback will be provided. Training sessions 2 and 4 are conceptualized as training on the job, including short inputs on interprofessional collaboration, the communication of prognosis and uncertainty. The main part of these sessions focus on workplace-based aspects that comprise an observation of the participating physicians and nurses during a milestone conversation with real patients. After the sessions trainers will give individualized feedback to training participants.

7 Where

Training sessions 1 and 3 will take place on the campus of the University of Heidelberg, Training 2 and 4 will take place in the hospital outpatient department of the Department of Thoracic Diseases at the University Hospital Heidelberg.

8 When and how much

Four sessions (6-8 hours each) with 3 to 4 weeks in between over a period of three months.

9 Tailoring

Depending on the results of participant questionnaires and interviews, training will be adapted to the needs and experiences of the training participants.

10 Modification

Not applicable

11 Adherence/fidelity (plan)

Observations during training sessions 2 and 4 using the training observation checklist

12 Adherence/fidelity (actual)

Not applicable

4.2 Milestone conversation

Planned and structured physician-nurse-patient conversations including caregivers at four points (milestones) within the disease trajectory:

5 Who

- Physicians who treat patients with a limited prognosis and who have to break bad news
- Registered nurses with a three-year vocational training, at least two years of clinical experience and a supplementary training in palliative care (German nursing education system)
- Patients with a prognosis of less than 12 months and
- The caregiver indicated by patient

6 How

Planned appointments of patient, caregiver, physician and nurse. Conversation according to the MCA manual and training. Consultations are developed on evidence-based literature [7-10] and on Spikes protocol [6], Tessmers description [11] and our work [12-14]

7 Where

Appointment in the out- or inpatient setting within the Department of Thoracic Oncology, University Hospital Heidelberg, depending on the disease trajectory. Milestone conversations will take place in a quiet, bright room with a Do-Not-Disturb sign on the door.

8 When and how much

Planned 30-minute appointments at the following four points within the disease trajectory:

- Milestone 1 "Diagnosis and Prognosis" within 8 weeks of estimated prognosis of less than 12 months
- Milestone 2 "stable phase in the course of treatment" 10 weeks after initiated treatments
- Milestone 3 "Progression of the disease" (e.g. tumor progression, FEV1 less than 30%, oxygen dependence, weight loss or cachexia)
- Milestone 4 "Transition to best supportive care" (e.g. lack of additional treatment options, no tumor-related treatment options, high symptom burden)

9 Tailoring

Not applicable

11 Adherence/Fidelity

Fidelity will be assessed using a content analysis of patient files and interviews and focus groups with nurses and physicians as described in Methods.

12 Adherence/fidelity (actual)

Not applicable

4.3 Follow-up session

Monthly follow-up sessions for patients and their caregivers by a nurse

5 Who

Nurse who has completed the MCA training and taken part in (was present at) the milestone conversation of a patient offers to call the patient and/or caregiver.

6 How

Follow-up session to assess communication needs, to assess palliative care needs (IPOS), to answer questions on diagnosis and therapy, to enhance prognostic awareness, to offer ACP and EPC and referral to specialists (e.g. dietician, psycho-oncologist, home health services, social services)

7 Where

Phone call (patient home) or personally if patient is in-house

8 When and how much

The nurse initially calls the patient one week after the first milestone conversation and then continues the calls on a monthly basis

9 Tailoring

Not applicable

10 Modifications

Not applicable

11 Adherence/Fidelity

Fidelity is assessed using a content analysis of patient files and interviews and focus groups with nurses and physicians as described in Methods.

12 Adherence/fidelity (actual)

Not applicable

References

1. Brandes K, Butow PN, Tattersall MH, Clayton JM, Davidson PM, Young J, et al. Advanced cancer patients' and caregivers' use of a Question Prompt List. Patient education and counseling. 2014;97(1):30-7.
2. Rodenbach RA, Brandes K, Fiscella K, Kravitz RL, Butow PN, Walczak A, et al. Promoting End-of-Life Discussions in Advanced Cancer: Effects of Patient Coaching and Question Prompt Lists. J Clin Oncol. 2017;35(8):842-51.
3. Clayton JM, Butow PN, Tattersall MH, Devine RJ, Simpson JM, Aggarwal G, et al. Randomized controlled trial of a prompt list to help advanced cancer patients and their caregivers to ask questions about prognosis and end-of-life care. J Clin Oncol. 2007;25(6):715-23.
4. Hearn J, Higginson IJ. Development and validation of a core outcome measure for palliative care: the palliative care outcome scale. Palliative Care Core Audit Project Advisory Group. Quality in health care : QHC. 1999;8(4):219-27.
5. Jackson VA, Jacobsen J, Greer JA, Pirl WF, Temel JS, Back AL. The cultivation of prognostic awareness through the provision of early palliative care in the ambulatory setting: a communication guide. Journal of palliative medicine. 2013;16(8):894-900.
6. Baile WF, Buckman R, Lenzi R, Glober G, Beale EA, Kudelka AP. SPIKES—A Six-Step Protocol for Delivering Bad News: Application to the Patient with Cancer. Oncologist. 2000;5:302-3011.
7. Hui D, Bruera E. Integrating palliative care into the trajectory of cancer care. Nature reviews Clinical oncology. 2016;13(3):159-71.
8. Pfeil TA, Laryionava K, Reiter-Theil S, Hiddemann W, Winkler EC. What keeps oncologists from addressing palliative care early on with incurable cancer patients? An active stance seems key. Oncologist. 2015;20(1):56-61.
9. Zimmermann C, Swami N, Krzyzanowska M, Hannon B, Leighl N, Oza A, et al. Early palliative care for patients with advanced cancer: a cluster-randomised controlled trial. Lancet. 2014;383(Mai 17):1723-30.
10. Temel JS, Greer JA, Muzikansky A, Gallagher ER, Admane S, Jackson VA, et al. Early Palliative Care for Patients with Metastatic Non–Small-Cell Lung Cancer. NEJM. 2010;363(8):733-42.
11. Tessmer G, Zaba O, Grohé C. Konzept einer vorausschauenden Kommunikation in der palliativen Behandlung von Patienten mit pneumolgisch-onkologischen Erkrankung. Pneumologie. 2011;65:503-9.
12. Sparla A, Villalobos M, Krug K, Kamradt M, Szecsenyi J, Thomas M, et al. Reflection of illness and strategies for handling advanced lung cancer : a qualitative analysis in patients and their relatives. BMC Health Services Research 2017;173(17).
13. Sparla A, Flach-Vorgang S, Villalobos M, Krug K, Kamradt M, Coulibaly K, et al. Individual difficulties and resources – a qualitative analysis in patients with advanced lung cancer and their relatives. 2016.
14. Villalobos M, Coulibaly K, Hermann K, Kamradt M, Wensing M, Siegle A, et al. A longitudinal communication approach in advanced lung cancer: a qualitative study of patients', relatives' and staff's perspectives European Journal of Cancer Care 2017: submitted
