# Supplementary material for: The Heidelberg Milestones Communication Approach (MCA) for patients with prognosis <12 months: protocol for a mixed-methods study including a randomized controlled trial
Source: Trials. 2018 Aug 14;19:438. doi: 10.1186/s13063-018-2814-1 (PMC6092809; doi:10.1186/s13063-018-2814-1)
Supplement: Supplementary file 2 — SPIRIT 2013 Checklist: Recommended items to address in a clinical trial protocol and related documents*. (PDF 144 kb) [file 13063_2018_2814_MOESM2_ESM.pdf]

SPIRIT 2013 Checklist: Recommended items to address in a clinical trial protocol and related documents*

Section/item	Item No	Description
**Administrative information**		
Title	1	Descriptive title identifying the study design, population, interventions, and, if applicable, trial acronym see page 1
Trial registration	2a	Trial identifier and registry name. If not yet registered, name of intended registry (Trial registration included) see page 3
	2b	All items from the World Health Organization Trial Registration Data Set (all items are described in the DRKS registry)
Protocol version	3	Date and version identifier (see footer)
Funding	4	Sources and types of financial, material, and other support (page 18)
Roles and responsibilities	5a	Names, affiliations, and roles of protocol contributors (see page 1)
	5b	Name and contact information for the trial sponsor (see page 18)
	5c	Role of study sponsor and funders, if any, in study design; collection, management, analysis, and interpretation of data; writing of the report; and the decision to submit the report for publication, including whether they will have ultimate authority over any of these activities (sponsor and funders have none of these roles)
	5d	Composition, roles, and responsibilities of the coordinating centre, steering committee, endpoint adjudication committee, data management team, and other individuals or groups overseeing the trial, if applicable (see Item 21a for data monitoring committee).(Not applicable)
**Introduction**		
Background and rationale	6a	Description of research question and justification for undertaking the trial, including summary of relevant studies (published and unpublished) examining benefits and harms for each intervention (included in Background) pages 3-4
	6b	Explanation for choice of comparators (included in Background) pages 3-4
Objectives	7	Specific objectives or hypotheses (included in Aims and Objective) page 4

Trial design	8	Description of trial design including type of trial (eg, parallel group, crossover, factorial, single group), allocation ratio, and framework (eg, superiority, equivalence, noninferiority, exploratory) (included in Abstract→Methods) pages 2 and 8

Methods: Participants, interventions, and outcomes

Study setting	9	Description of study settings (eg, community clinic, academic hospital) and list of countries where data will be collected. Reference to where list of study sites can be obtained (included in Data Collection) page 8
Eligibility criteria	10	Inclusion and exclusion criteria for participants. If applicable, eligibility criteria for study centres and individuals who will perform the interventions (eg, surgeons, psychotherapists) (included in Setting: eligibility criteria for study centres and individuals performing inventions (page 8); included in Study Population: inclusion and exclusion criteria for participants (page 10))
Interventions	11a	Interventions for each group with sufficient detail to allow replication, including how and when they will be administered (included in Development of the Heidelberg Milestone Communication Approach (MCA)) pages 5-7, (included in Measures) pages 8-9, included in Additional File 1 intervention description according to TiDieR)
	11b	Criteria for discontinuing or modifying allocated interventions for a given trial participant (eg, drug dose change in response to harms, participant request, or improving/worsening disease) (Criteria for discontinuing are new results from other studies, making this study unnecessary. There are no modifying allocated interventions – once one milestone conversation has taken place, patient data will remain in the control group. Any patient wish for no further intervention will be respected)
	11c	Strategies to improve adherence to intervention protocols, and any procedures for monitoring adherence (eg, drug tablet return, laboratory tests) (are part of the study and described in PDCA (Plan-Do-Check-Act-) cycle) see page 8
	11d	Relevant concomitant care and interventions that are permitted or prohibited during the trial (not applicable)
Outcomes	12	Primary, secondary, and other outcomes, including the specific measurement variable (eg, systolic blood pressure), analysis metric (eg, change from baseline, final value, time to event), method of aggregation (eg, median, proportion), and time point for each outcome. Explanation of the clinical relevance of chosen efficacy and harm outcomes is strongly recommended (included in Outcome Evaluation) pages 9-10

Participant timeline	13	Time schedule of enrolment, interventions (including any run-ins and washouts), assessments, and visits for participants. A schematic diagram is highly recommended (see Figure) (included in Development of the Heidelberg Milestone Communication Approach (MCA)) pages 5-7, schematic diagram see Additional File.
Sample size	14	Estimated number of participants needed to achieve study objectives and how it was determined, including clinical and statistical assumptions supporting any sample size calculations (included in Study Population) page 1; (clinical and statistical assumptions supporting any sample size calculations included in Power Calculation) page 13; Additional sample size for interviews page 14 and interprofessional collaboration page 15
Recruitment	15	Strategies for achieving adequate participant enrolment to reach target sample size (included in Study Population) page 10

Methods: Assignment of interventions (for controlled trials)

Allocation:

Sequence generation	16a	Method of generating the allocation sequence (eg, computer-generated random numbers), and list of any factors for stratification. To reduce predictability of a random sequence, details of any planned restriction (eg, blocking) should be provided in a separate document that is unavailable to those who enrol participants or assign interventions (included in Randomization Procedure) page 11
Allocation concealment mechanism	16b	Mechanism of implementing the allocation sequence (eg, central telephone; sequentially numbered, opaque, sealed envelopes), describing any steps to conceal the sequence until interventions are assigned (included in Randomization Procedure) page 11
Implementation	16c	Who will generate the allocation sequence, who will enrol participants, and who will assign participants to interventions (included in Implementation) pages 8-15
Blinding (masking)	17a	Who will be blinded after assignment to interventions (eg, trial participants, care providers, outcome assessors, data analysts), and how (no blinding)
	17b	If blinded, circumstances under which unblinding is permissible, and procedure for revealing a participant's allocated intervention during the trial (no blinding)

Methods: Data collection, management, and analysis

Data collection methods	18a	Plans for assessment and collection of outcome, baseline, and other trial data, including any related processes to promote data quality (eg, duplicate measurements, training of assessors) and a description of study instruments (eg, questionnaires, laboratory tests) along with their reliability and validity, if known. Reference to where data collection forms can be found, if not in the protocol (included in Measures) pages 8-9; (forms included in instruments) pages 10-12; additional data collection and analysis/instruments for interviews page 13-14 and interprofessional collaboration page 14-15
	18b	Plans to promote participant retention and complete follow-up, including list of any outcome data to be collected for participants who discontinue or deviate from intervention protocols (included in data collection) page 10, study nurse ensured participant retention and complete follow-up; only the number of participants who discontinued will be collected.
Data management	19	Plans for data entry, coding, security, and storage, including any related processes to promote data quality (eg, double data entry; range checks for data values). Reference to where details of data management procedures can be found, if not in the protocol (included in Ethical Aspects) page 16
Statistical methods	20a	Statistical methods for analysing primary and secondary outcomes. Reference to where other details of the statistical analysis plan can be found, if not in the protocol (included in Power Calculation) page 13 (and included in Data Analysis) pages 14 and 16
	20b	Methods for any additional analyses (eg, subgroup and adjusted analyses) (additional analysis for interviews and interprofessional collaboration) pages 14-15
	20c	Definition of analysis population relating to protocol non-adherence (eg, as randomised analysis), and any statistical methods to handle missing data (eg, multiple imputation) (included in data analysis) pages 12-13
**Methods: Monitoring**		
Data monitoring	21a	Composition of data monitoring committee (DMC); summary of its role and reporting structure; statement of whether it is independent from the sponsor and competing interests; and reference to where further details about its charter can be found, if not in the protocol. Alternatively, an explanation of why a DMC is not needed (not applicable)
	21b	Description of any interim analyses and stopping guidelines, including who will have access to these interim results and make the final decision to terminate the trial (not applicable)

Harms	22	Plans for collecting, assessing, reporting, and managing solicited and spontaneously reported adverse events and other unintended effects of trial interventions or trial conduct (patient documents will be checked by the study nurse for adverse events; no adverse events anticipated) page 11
Auditing	23	Frequency and procedures for auditing trial conduct, if any, and whether the process will be independent from investigators and the sponsor (Not applicable)
**Ethics and dissemination** (included in Ethics Aspects) page 16		
Research ethics approval	24	Plans for seeking research ethics committee/institutional review board (REC/IRB) approval page 16
Protocol amendments	25	Plans for communicating important protocol modifications (eg, changes to eligibility criteria, outcomes, analyses) to relevant parties (eg, investigators, REC/IRBs, trial participants, trial registries, journals, regulators) (no plans for changes at the moment)
Consent or assent	26a	Who will obtain informed consent or assent from potential trial participants or authorised surrogates, and how (see Item 32) page 10
	26b	Additional consent provisions for collection and use of participant data and biological specimens in ancillary studies, if applicable (not applicable)
Confidentiality	27	How personal information about potential and enrolled participants will be collected, shared, and maintained in order to protect confidentiality before, during, and after the trial page 16 (only the study nurse will have a list with the number of eligible participants and non-responders. Only the study nurse will know the names of the participating patients).
Declaration of interests	28	Financial and other competing interests for principal investigators for the overall trial and each study site page 18
Access to data	29	Statement of who will have access to the final trial dataset, and disclosure of contractual agreements that limit such access for investigators (only investigators will have access to final trial dataset; there are no contractual agreements that limit such access) page 16
Ancillary and post-trial care	30	Provisions, if any, for ancillary and post-trial care, and for compensation to those who suffer harm from trial participation (referral to psycho-oncology services if there is any harm; no harm is anticipated) page 6

Dissemination policy	31a	Plans for investigators and sponsor to communicate trial results to participants, healthcare professionals, the public, and other relevant groups (eg, via publication, reporting in results databases, or other data sharing arrangements), including any publication restrictions (at the end of the study, there will be a symposium for the public, publication of results in journal and report to funders)
	31b	Authorship eligibility guidelines and any intended use of professional writers (not applicable)
	31c	Plans, if any, for granting public access to the full protocol, participant-level dataset, and statistical code (not applicable)
**Appendices** (not applicable)		
Informed consent materials	32	Model consent form and other related documentation given to participants and authorised surrogates
Biological specimens	33	Plans for collection, laboratory evaluation, and storage of biological specimens for genetic or molecular analysis in the current trial and for future use in ancillary studies, if applicable

*It is strongly recommended that this checklist be read in conjunction with the SPIRIT 2013 Explanation & Elaboration for important clarification on the items. Amendments to the protocol should be tracked and dated. The SPIRIT checklist is copyrighted by the SPIRIT Group under the Creative Commons "Attribution-NonCommercial-NoDerivs 3.0 Unported" license.
