# Supplementary material for: The Heidelberg Milestones Communication Approach (MCA) for patients with prognosis <12 months: protocol for a mixed-methods study including a randomized controlled trial
Source: Trials. 2018 Aug 14;19:438. doi: 10.1186/s13063-018-2814-1 (PMC6092809; doi:10.1186/s13063-018-2814-1)
Supplement: Supplementary file 3 — Confirmation Approval. (PDF 717 kb) [file 13063_2018_2814_MOESM3_ESM.pdf]

Medizinische Fakultät Heidelberg

Ethikkommission der Med. Fak. HD | Alte Glockengießerei 11/1 | D-69115 Heidelberg

Herrn Matthias Villalobos
Internistische Onkologie Thorakaler Tumoren
Thoraxklinik
Universität Heidelberg
Röntgenstrasse 1
69126 Heidelberg

23.03.2018
ts-cd

Confirmation Approval

| | |
|---|---|
| *Unser Zeichen:* | 561/2017 (Bitte stets angeben) |
| *Titel:* | Evaluation des Heidelberger Meilenstein-Kommunikationskonzeptes (HeiMeKOM) Phasen 2 und 3 |
| *Eingereichte Unterlagen:* | <u>Ersteinreichung vom 12.10.2017:</u><br>Anschreiben vom 12.10.2017<br>Zusammenfassung Version 1.00 vom 12.10.2017<br>Checkliste Sonstige Studien<br>Formular für Erstantrag<br>Informationsschreiben (für Untersuchungen im Klinikteam zum Forschungsprojekt) Version 1.0<br>Informationsschreiben (für qualitative Untersuchungen mit Ärzten und Pflegekräften zum Forschungsprojekt) Version 1.0<br>Informationsschreiben (für Patienten und Angehörige zu Gesprächen) Version 1.0<br>Informationsschreiben (für Patienten/Angehörige zum Forschungsprojekt) Version 1.0<br>Informationsschreiben (zur Evaluation der Schulung im Forschungsprojekt) Version 1.0<br>Informationsschreiben (für Patienten zur Videoaufzeichnung des Gespräches mit Arzt und Pflege) Version 1.0<br>Einwilligungserklärung (für Untersuchungen im Klinikteam) Version 1.0<br>Einwilligungserklärung (für qualitative Untersuchungen mit Ärzten und Pflegekräften) Version 1.0<br>Einwilligungserklärung (für Patienten und Angehörigen zu Gesprächen) Version 1.0<br>Einwilligungserklärung (für Patienten/Angehörige) Version 1.0<br>Einwilligungserklärung (zur Evaluation der Schulung) Version 1.0<br>Einwilligungserklärung (für Patienten zur Videoaufzeichnung des Gesprächs mit Arzt und Pflege) Version 1.0<br>Studienprotokoll Version 1.0 vom 12.10.2017<br>CV Matthias Villalobos vom 12.10.2017<br><u>Inhaltliche Nachreichung vom 23.11.2017:</u><br>Ihr Anschreiben vom 23.11.2017<br>Informationsschreiben (für Untersuchungen im Klinikteam zum Forschungsprojekt) Version 1.1 (mit Markierung der Änderungen)<br>Informationsschreiben (für qualitative Untersuchungen mit Ärzten und Pflegekräften zum Forschungsprojekt) Version 1.1 (mit Markierung der Änderungen)<br>Informationsschreiben (für Patienten zu Gesprächen) Version 1.1 (mit Markierung der Änderungen) |

Ethikkommission
Medizinische Fakultät Heidelberg

Alte Glockengießerei 11/1
D-69115 Heidelberg

☏ +49 (0) 6221 / 33 8 22 0 (Empfang)
🖷 +49 (0) 6221 / 33 8 22 22
ethikkommission-I@med.uni-heidelberg.de

www.medizinische-fakultaet-hd.uni-heidelberg.de/ethikkommission

**Vorsitz:**
Prof. Dr. med. Thomas Strowitzki

**Stellv. Vorsitz:**
Prof. Dr. med. Johannes Schröder
Prof. Dr. med. Klaus Herfarth

**Geschäftsleitung:**
Dr. med. Verena Pfeilschifter

**Sonstige Studien:**
Gero Blim, LL.M.
☏ +49 6221 3382218
🖷 +49 6221 3382222
Gero.Blim@med.uni-heidelberg.de

Christian Deisenroth, M.A.
☏ +49 6221 3382215
🖷 +49 6221 3382222
Christian.Deisenroth@med.uni-heidelberg.de

Dr. rer. nat. Sabine Vogel
☏ +49 6221 3382219
🖷 +49 6221 3382222
Sabine.Vogel@med.uni-heidelberg.de

Qualitätsmanagement ISO 9001:2015
399893 QM 15

**Bankverbindung:**
Baden-Württembergische Bank Stuttgart
Konto-Nr.: 7421 500 429
BLZ: 600 501 01
SWIFT/BIC Code: SOLADEST
IBAN-Nr.: DE 64600501017421500429

Informationsschreiben (für Patienten zum Forschungsprojekt) Version 1.1 (mit Markierung der Änderungen)
Informationsschreiben (für Mitarbeiter zur Evaluation der Schulung im Forschungsprojekt) Version 1.1 (mit Markierung der Änderungen)
Informationsschreiben (für Patienten zur Videoaufzeichnung des Gespräches mit Arzt und Pflege) Version 1.1 (mit Markierung der Änderungen)
Informationsschreiben für Angehörige zu Gesprächen Version 1.0 (neues Dokument)
Informationsschreiben für Angehörige zur Videoaufzeichnung mit Arzt und Pflege Version 1.0 (neues Dokument)
Einwilligungserklärung (für Untersuchungen im Klinikteam) Version 1.1 (mit Markierung der Änderungen)
Einwilligungserklärung (für qualitative Untersuchungen mit Ärzten und Pflegekräften) Version 1.1 (mit Markierung der Änderungen)
Einwilligungserklärung (für Patienten und Angehörigen zu Gesprächen) Version 1.1 (mit Markierung der Änderungen)
Einwilligungserklärung (für Patienten) Version 1.1 (mit Markierung der Änderungen)
Einwilligungserklärung (für Mitarbeiter zur Evaluation der Schulung) Version 1.1 (mit Markierung der Änderungen)
Einwilligungserklärung (für Patienten zur Videoaufzeichnung des Gesprächs mit Arzt und Pflege) Version 1.1 (mit Markierung der Änderungen)
Einwilligungserklärung für Angehörige zu Gesprächen Version 1.0 (neues Dokument)
Einwilligungserklärung für Angehörige zur Videoaufzeichnung des Gesprächs mit Arzt und Pflege Version 1.0 (neues Dokument)
Studienprotokoll Version 1.1 vom 23.11.2017 (mit Markierung der Änderungen)
Gesundheitsfragebogen für Patienten (PHQ-4)
Fragebogen NCCN Distress-Thermometer
Fragebogen FACT-G (Fassung 4)
Fragebogen FACT-L (Fassung 4)
Fragebogen zum Unterstützungsbedarf von Angehörigen Krebskranker SCNS P&C-G
Fragebogen SEIQoL-Q
The UWE (University of the West of England) Interprofessional Questionnaire

Dear Mr. Villalobos

we hereby confirm that the above mentioned research project has been reviewed and approved by the Ethics Committee of the Medical Faculty of Heidelberg University. The approval of the Ethics Committee (Berufsrechtliche Beratung), dated to November 29 2017, has been sent to you on November 29 2017.

Kind regards

i.V.

Digital unterschrieben von Dr. Pfeilschifter, Verena
DN: c=DE, sn=Pfeilschifter, givenName=Verena, cn=Dr. Pfeilschifter, Verena, serialNumber=1
Datum: 2018.03.23 10:05:48 +01'00'

Prof. Dr. med. Thomas Strowitzki
Vorsitzender
